# Supplementary material for: FERN – a Java framework for stochastic simulation and evaluation of reaction networks
Source: BMC Bioinformatics. 2008 Aug 29;9:356. doi: 10.1186/1471-2105-9-356 (PMC2553347; doi:10.1186/1471-2105-9-356)
Supplement: Additional file 1 — FERN distribution, Version 1.3. This archive contains the FERN source code and binaries as well as documentation and example models in FernML and SBML. [file 1471-2105-9-356-S1.zip › fern/doc/javadoc/fern/simulation/controller/class-use/SimulationController.html]

Uses of Interface fern.simulation.controller.SimulationController


---


|  |  |  |
| --- | --- | --- |
| **Overview** \| **Package** \| **Class** \| **Use** \| **Tree** \| **Deprecated** \| **Index** \| **Help** | |  |
| PREV   NEXT | **FRAMES**    **NO FRAMES**     **All Classes** |  |


---


## **Uses of Interface fern.simulation.controller.SimulationController**

| Packages that use SimulationController | |
| --- | --- |
| **fern.benchmark** | Provides classes for benchmarking simulations (time benchmarks, histogram distance calculation). |
| **fern.simulation** | Provides algorithms for simulating reaction network as well as methods for observer certain aspects of a simulation. |
| **fern.simulation.algorithm** | Provides algorithms for stochastic simultion of reaction networks. |
| **fern.simulation.controller** | Provides classes to control the simulations. |
| **fern.simulation.observer** | Provides classes to observe certain aspects of simulations. |

Uses of SimulationController in fern.benchmark

| Methods in fern.benchmark that return SimulationController | |
| --- | --- |
| `protected  SimulationController` | `SimulatorTime.getController(int i)`             Returns the `SimulationController` for the base class. |
| `protected  SimulationController` | `SimulatorRandomNumbers.getController(int i)`             Returns the `SimulationController` for the base class. |
| `protected abstract  SimulationController` | `SimulatorPerformance.getController(int i)`             Extending classes have to determine the `SimulationController` of each `Simulator` here. |
| `protected  SimulationController` | `SimulatorCorrectness.getController(int i)`             Returns the `SimulationController` for the base class. |

Uses of SimulationController in fern.simulation

| Methods in fern.simulation with parameters of type SimulationController | |
| --- | --- |
| `abstract  void` | `Simulator.performStep(SimulationController control)`             Performs one simulation step. |
| `void` | `Simulator.start(SimulationController control)`             Start the simulation with the given `SimulationController`. |

Uses of SimulationController in fern.simulation.algorithm

| Methods in fern.simulation.algorithm with parameters of type SimulationController | |
| --- | --- |
| `void` | `HybridMaximalTimeStep.performStep(SimulationController control)` |
| `void` | `GillespieSimple.performStep(SimulationController control)` |
| `void` | `GillespieEnhanced.performStep(SimulationController control)` |
| `void` | `GibsonBruckSimulator.performStep(SimulationController control)` |
| `void` | `AbstractBaseTauLeaping.performStep(SimulationController control)`             Performs a tau leaping step. |

Uses of SimulationController in fern.simulation.controller

| Classes in fern.simulation.controller that implement SimulationController | |
| --- | --- |
| `class` | `AndController`             Implements a `SimulationController` composed of many `SimulationController`. |
| `class` | `DefaultController`             The probably most common implementation of an `SimulationController`. |
| `class` | `OrController`             Implements a `SimulationController` composed of many `SimulationController`. |

Constructors in fern.simulation.controller with parameters of type SimulationController

`AndController(SimulationController... controllers)`

`OrController(SimulationController... controllers)`

Uses of SimulationController in fern.simulation.observer

| Classes in fern.simulation.observer that implement SimulationController | |
| --- | --- |
| `class` | `AmountAtMomentObserver`             Observes the amount of some molecule species at a certain moment. |

---


|  |  |  |
| --- | --- | --- |
| **Overview** \| **Package** \| **Class** \| **Use** \| **Tree** \| **Deprecated** \| **Index** \| **Help** | |  |
| PREV   NEXT | **FRAMES**    **NO FRAMES**     **All Classes** |  |


---
